# Interpreting a Bayesian phase II futility clinical trial

**DOI:** 10.1186/s13063-022-06877-7

**Published:** 2022-11-22

**Authors:** Jonathan Beall, Christy Cassarly, Renee Martin

**Affiliations:** Department of Public Health Sciences, Medical University of South Carolina, Charleston, SC USA

**Keywords:** Bayesian, Phase II, Futility, Clinical trial

## Abstract

**Background:**

A resurgence of research into phase II trial design in the mid-2000s led to the use of futility designs in a wide variety of disease areas. Phase II futility studies differ from efficacy studies in that their null hypothesis is that treatment, relative to control, does not meet or exceed the level of benefit required to justify additional study. A rejection of the null hypothesis indicates that the treatment should not proceed to a larger confirmatory trial.

**Methods:**

Bayesian approaches to the design of phase II futility clinical trials are presented and allow for the quantification of key probabilities, such as the predictive probability of current trial success or even the predictive probability of a future trial’s success.

**Results:**

We provide an illustration of the design and interpretation of a phase II futility study constructed in a Bayesian framework. We focus on the operating characteristics of our motivating trial based on a simulation study, as well as the general interpretation of trial outcomes, type I, and type II errors in this framework.

**Conclusions:**

Phase II futility clinical trials, when designed under in a Bayesian framework, offer an alternative approach to the design of mid-phase studies which provide unique benefits relative to trials designed in a frequentist framework and designs which focus on treatment efficacy.

## Background

### Phase II futility studies

Phase II clinical trials that focused on determining whether or not a treatment warrants additional research have been a mainstay of the clinical trialist’s toolkit since their initial development in the 1960s. While initially developed for oncology-focused trials, a resurgence of research into phase II designs in the mid-2000s led to the use of futility-based phase II clinical trial designs being found in a wide variety of disease areas [1]. Researchers have noted the utility of futility trials as a screening tool to help guide clinical research away from treatments with a poor likelihood of success in the larger phase III setting as the goal of a phase II trial is to determine whether or not additional research into the study drug is futile [2, 3].

Phase II futility (or non-superiority) studies differ from efficacy-based studies in that their null hypothesis is that treatment, relative to control, does not meet or exceed the necessary level of benefit required to justify additional study. Given the alternative hypothesis is that the treatment is futile when compared to the control, a rejection of the null hypothesis indicates the treatment should not proceed to a larger confirmatory trial. It is common for a phase II futility trial to be designed as a single-arm study, with comparisons made to a pre-defined outcome rate in patients treated with the standard of care, for example. However, for the purposes of our work, we will focus on a trial design with two treatment groups to which subjects are randomized.

One example of the null and alternative hypotheses of a futility trial are $H_0: \pi _t - \pi _c \ge \Delta$ and $H_1: \pi _t - \pi _c < \Delta$, where $\Delta$ is the margin of superiority and $\pi _c$ and $\pi _t$ are the probability of a favorable outcome for the control and treatment arms, respectively [1, 2]. Futility in this case simply means that the observed difference in proportions does not exceed that of the margin of superiority (see Table 1 for example interpretations of trial outcomes). This margin, fundamental to the construction of the trial, can be characterized as the minimal difference required for the treatment to be considered worthy of additional study [2]. For single-arm studies, $\pi _c$ is a fixed quantity whereas in a multi-arm trial $\pi _c$ is estimated from a concurrent control group.

**Table 1:** Example interpretations of outcomes for phase II futility trial

	Phase II futility	Phase II efficacy
Fail to reject $H_0$	The data do not rule out superiority of the new treatment relative to control.	There is not sufficient evidence that the treatment is efficacious relative to control and therefore does not warrant additional review.
Reject $H_0$	There is sufficient evidence that the benefit of the treatment is less than desired and it is futile to proceed to phase III.	The treatment demonstrates a signal of improvement relative to control and therefore warrants additional study.

Traditional interpretations of a trial which fails to reject the null hypothesis under an efficacy-based design are negative, as the treatment has failed to prove itself relative to control; however, the interpretation changes in the described phase II futility trial setting. A futility trial with an inability to reject the null hypothesis indicates a treatment for which there is insufficient evidence of futility, a positive indication towards an efficacious treatment. Levin clarifies this statement, insisting that in this setting we cannot accept the null hypothesis; however, we can conclude that “the data do not rule out superiority” [1]. Similarly, in an efficacy-based design, a trial with enough evidence to reject the null hypothesis represents a positive clinical finding. However, in the described phase II futility setting, a trial which rejects the null hypothesis indicates a treatment for which futility is likely given the current data, a negative clinical finding.

Similar to the interpretations of the trial outcome, outcome errors also have altered interpretations under a phase II futility trial relative to an efficacy-based trial. A type I error in the phase II futility setting occurs when a treatment is declared futile when in fact the treatment does provide benefit relative to control. A type I error would prevent a treatment which warrants additional study from proceeding to a larger confirmatory setting. Committing a type II error in the phase II futility setting is a futile treatment failing to be declared as such. Here, a type II error could lead to a future trial being planned and executed for a treatment which does not warrant an additional study (Table 2).

**Table 2:** Interpretation of type I and type II errors

	Should not reject $H_0$	Should reject $H_0$
Fail to reject $H_0$	No error	Type II error—we are unable to rule out the futility of a treatment for which futility should be declared.
Reject $H_0$	Type 1 error—we label a treatment as futile relative to control which should not be labeled as futile.	No error

Novel phase II trial designs are abundant in the literature, with many of these innovations focusing on adaptations and implementations of a frequentist single-arm approach or Bayesian adaptations of multi-arm trials [3–7]. Early research into phase II futility designs focused on Bayesian and frequentist methodologies for oncology clinical trials, with later research extending these methodologies to other disease areas such as stroke [1, 8–11]. Bayesian approaches to the design and conduct of clinical trials have been noted as having several benefits relative to their frequentist counterparts. First, Bayesian inference does not require results to be conditioned upon the null hypothesis being true; rather, results are interpreted as being conditioned on the observed data. That is, a Bayesian approach can quantify the probability of the alternative hypothesis being true given the observed data whereas a frequentist *p*-value represents the probability of observing data as or more extreme than the observed data given the null hypothesis is true [12]. This difference results in a direct and interpretable response to clinically meaningful questions [12–14]. Bayesian methods also allow for the incorporation of prior information when quantifying the likelihood of an outcome, can avoid penalties that are associated with frequentist approaches to interim monitoring during trials, appropriately address the variability invoked when estimating parameters by assuming parameters are associated with a distribution, and allow for the estimation of the uncertainty associated with parameters and quantities of interest [12–16]. In the context of clinical trials, Bayesian approaches can also be viewed as superior to frequentist approaches with respect to a clinical trial acting as a decision-making process. Following the explanation of Berry, there are numerous decisions which can occur before, during, or after a clinical trial [16]. Bayesian methods allow for these decisions to be made using results drawn from the available information, such as the predictive probability of current trial success or even the predictive probability of a future trial’s success [16]. The ability to construct these and other predictive probabilities provide a substantive argument for the utilization of Bayesian methods over frequentist approaches [17]. However, while there are numerous benefits to Bayesian designs, there are potential drawbacks. Specifically, for clinical trials, the utilization of prior information can be viewed as problematic by governing and regulatory boards should the prior information utilized be too strong. In this setting, careful consideration must be given to the utilization of prior information and the construction of the prior distribution.

This work was motivated by requests for an adaptation of the frequentist futility trial design which would improve in the interpretations of findings and increase the flexibility of the chosen analytic model without degrading the statistical design properties. Given the benefits of Bayesian clinical trial designs detailed in the literature, the extension and implementation of a phase II futility clinical trial in a Bayesian framework would allow for the desired alterations to the standard futility design.

## Methods

Consider a hypothetical multicenter, randomized, double-blind phase II futility trial evaluating the use of a new treatment for aneurysmal subarachnoid hemorrhage (aSAH) and whether or not it is worthwhile to move to this treatment forward to a phase III efficacy trial. For our hypothetical trial, we define the probability of a favorable outcome $\pi _d$, where *d* reflects the treatment arm with $d \in (c,t)$. We say that the number of favorable outcomes in treatment arm *d*, noted $Y_d$, follows a binomial distribution with $n_d$ trials and probability $\pi _d$. That is, $Y_d \sim {Binomial}(n_d,\pi _d)$, with $n_d$ representing the number of subjects randomized to treatment arm *d*. The total sample size for the trial is 500 subjects with equal allocation to both treatment arms and with the margin of superiority $(\Delta )$ set to 0.09. We now provide a simulation study in the context of our motivating trial.

### Bayesian quantities of interest

The probability of outcome, $\pi _d$, is estimated using the Bayesian independent dose model from FACTS for each of the treatment arms as shown below in Eqs. 1 through 3 [18].

1 $$\begin{aligned} Y_d\sim & {} \text {Binomial}(n_d,\pi _d)\end{aligned}$$

2 $$\begin{aligned} \pi _d= & {} \frac{e^{\theta _d}}{1+e^{\theta _d}}\end{aligned}$$

3 $$\begin{aligned} \theta _d\sim & {} N\left( \mu _d,\sigma ^2_d\right) \end{aligned}$$

The independent dose model was chosen for this example as it is a simplistic approach which allows for the clear comparison of treatment arms. For this model, the log odds of outcome in each treatment arm, $\theta _d$, are given normal prior distributions with mean $\mu _d$ and standard deviation $\sigma _d$. For this work, $\mu _d = 0$ and $\sigma _d = 2$. This approach is useful for comparisons between treatment and control arms when there are a small set of treatment arms and there is no natural ordering of the treatment arms [18]. As an example of the flexibility afforded by Bayesian methods, should data exist from a prior study, i.e., from an earlier phase I clinical trial, the parameters of the prior distribution could be altered in order to incorporate this information when estimating the treatment effect in the current trial.

#### Posterior probability of treatment futility

The posterior probability of treatment futility is $P(\pi _t -\pi _c < \Delta |Y_t, Y_c, n_t,n_c)$. This posterior probability is conditioned on the observed data and quantifies the likelihood of the observed treatment effect being less than the pre-specified margin of superiority. This utilization of this posterior probability allows for a direct quantification of the clinical question of interest. In comparison to a frequentist approach to the phase II futility clinical trial, where the resultant *p*-value for the test of futility would reflect the likelihood of observing the current data assuming the null hypothesis is true, the construction of the posterior probability as a quantity of interest demonstrates the increased interpretability afforded to Bayesian methods relative to frequentist approaches.

#### Predictive probability of success in a future phase III trial

Given the role of the phase II futility trial as a screening tool to remove candidate treatments which are unlikely to be successful in a larger confirmatory trial, quantifying the likelihood of success in such a larger, future trial would provide meaningful insight during the trial planning period. For a given sample size and type 1 error rate of a future trial, we can calculate the posterior predictive probability of success by averaging the expected power for such a future trial across the posterior distribution. Here, the expected power is calculated for each MCMC sample and utilizes the estimates of response in each arm and the subsequent error associated with the estimates  [5, 18].

### Simulation study

For our simulation study, we will mimic the characteristics of the hypothetical trial; that is, we will have a total sample size of 500 subjects with equal allocation to 2 treatment arms. The margin of superiority, $\Delta$, will be set to 0.09, with the true treatment effect being one of 2 values: 0 or 0.09. The margin of superiority and total sample size were chosen to reflect a clinically relevant minimal clinically important difference and the feasible sample size of the proposed trial, respectively. In the context of a phase II futility trial, when the treatment is truly not superior when compared to the standard of care, we are able to characterize the power of the trial. Similarly, when the treatment is truly superior relative to the standard of care, we are able to characterize the type 1 error rate for the trial.

At the final analysis, a trial will be deemed successful if $P(\pi _t -\pi _c < \Delta | Y_t, Y_c, n_t, n_c)>0.90$. That is, if $P(\pi _t -\pi _c < \Delta |Y_t, Y_c, n_t, n_c)>0.90$, we will declare the benefit of the treatment arm is less than desired and it is futile to proceed to phase III. For each treatment arm in each simulation, we will also estimate the probability of success for a future efficacy-based trial. For this future trial, assuming a frequentist design to detect a difference of 0.09 between the treatment and standard of care arms with 80% power and a 2.5% one-sided type 1 error rate, this future phase III trial would require 882 patients with equal allocation to the treatment and standard of care arms. These characteristics are used in the calculation of the predictive probability of success for each treatment arm in each of our simulations.

Simulations to characterize trial performance will be conducted using the Fixed and Adaptive Clinical Trial Simulation (FACTS) software version 6.3. FACTS is a user-friendly program which allows for the quick construction and simulation of numerous Bayesian trial designs [18]. FACTS implements a Gibbs structure for the $\theta _d$ conditional on outcomes with sequential sampling from the conditional distribution for each of the $\theta _d$. Sampling for the complete conditional distribution of each $\theta _d$ is attained using a Metropolis-Hastings algorithm. Data are generated within FACTS, with the response rates for each simulated treatment arm specified by the user. Using the FACTS software, a total of 1000 simulations will be conducted per scenario, with each simulation having 1000 samples for burn-in, a total of 2500 samples, and no thinning of the samples. Given the simplicity of the model, as well as the model sampling approach, for this work, it is expected all models will converge and additional studies aimed at assessing convergence, mixing, and sensitivity of the model remain as future work.

## Results

Operating characteristics for our motivating trial are shown and interpreted in Table 3. The power and type 1 error rates are 80.5% and 9.2%, respectively. In the phase II futility setting, committing a type 1 error is more egregious than in the phase III setting as a type 1 error would prevent an efficacious treatment from moving to the phase III setting. While we want to avoid excessive type 1 errors when conducting a phase II futility trial, these trials are often designed with target type 1 error rates of 10%. These rates are set in order to prevent sample sizes from increasing beyond a justifiable level [2].

**Table 3:** Operating characteristics with interpretations

	Data simulated under null hypothesis	Data simulated under alternative hypothesis
Proportion successful trials	The probability of declaring a treatment futile, which is in fact not futile, is 9.2%	The probability of declaring a futile treatment futile is 80.5%.
Probability of success in phase III trial	The probability of a treatment, for which we cannot rule out superiority, achieving a significant result in a larger phase III efficacy-based trial is 68.64%.	The probability of a treatment, for which we cannot rule out superiority, achieving a significant result in a larger phase III efficacy-based trial is 17.8%.

## Conclusions

In this paper, we provide an illustration for the design and interpretation of a phase II futility study designed in a Bayesian framework. As previously discussed, Bayesian approaches demonstrate several benefits including increased interpretability when addressing the primary research question. These designs also allow for the quantification of probabilities which can be used when planning future trials, such as the predictive probability of success for a future efficacy-based trial. Another notable benefit of a Bayesian design is the ability to often avoid penalties when including interim analyses.

Bayesian adaptation of the phase II futility design presents an opportunity for a trial to be able to screen out unsuccessful treatments all while experiencing the benefits afforded by a Bayesian approach. In the context of the phase II futility study, the inclusion of interim analyses could allow for either the detection of a futile treatment earlier in the study or the earlier detection of the inability to show treatment futility. The former would further expand upon the goal of the futility study to act as a screening tool to remove unsuccessful treatments whereas the latter would allow a faster progression to a larger confirmatory study.

## Data Availability

Not applicable.
